# Application and impact assessment of an interactive journal club format among endocrinology fellows-in-training in a tertiary hospital academic center: a case study

**DOI:** 10.12688/mep.19740.1

**Published:** 2023-09-08

**Authors:** Harold Henrison Chiu, Iris Thiele Isip-Tan

**Affiliations:** 1 Division of Endocrinology, Diabetes and Metabolism, Department of Medicine, Philippine General Hospital, University of the Philippines Manila, Manila, Metro Manila, 1006, Philippines

**Keywords:** Interactive journal club, active learning, active listening, appraisal, critical thinking, medical education, medical training, endocrinology

## Abstract

**Background:** The interactive journal club is designed to utilize a new approach in appraising research in order to maximize the benefits of the traditional journal club. In this new approach, the participants are actively involved in a structured process of critical data appraisal rather than just being passive listeners. In this case study, we applied the interactive journal club format and assessed its impact among our endocrinology fellows-in-training.

**Methods:** We conducted four interactive journal club sessions within a four-week span, one per each week *via* a virtual platform. The 12 participants were the same throughout all sessions. Each session was recorded following informed consent. At the end of all sessions, feedback was obtained, tabulated and compared.

**Results:** Sessions lasted from 59 to 83 minutes (mean, 67.75 minutes). Participants became more active and spontaneous as the sessions progressed. All participants found the format more fun and proactive. This approach allowed more critical thinking and processing of information. Salient features include increased self-esteem and confidence, additional learning from other participants, better retention of information, and utilization in future practice.

**Conclusions:** Traditional approaches are transformed from passive presentations of recent developments in medicine into an interactive discussion while allowing the retention of the spirit and essence of a traditional journal club, as well as exploring new and improved approaches in clinical training and education.

## Introduction

Journal clubs have remained a staple activity in contemporary postgraduate clinical education. Since their formal inception in 1889 by Sir William Osler, journal clubs have the common goal of creating an avenue where individuals with a shared common interest can meet regularly to discuss ideas and new developments in medical literature ( McGlacken-Byrne *et al.*, 2020). Since then, journal clubs have evolved overtime and have given modern-day doctors good opportunities to discuss clinical dilemmas, treatment updates, management guidelines, consensus statements, and clinical decision making ( Aronson, 2017; McGlacken-Byrne *et al.*, 2020). The traditional journal club usually occurs face-to-face with a dedicated time and venue where peer-reviewed articles, novel and landmark studies are presented by trainees. These sessions are often moderated by a senior member of the team or an assigned faculty followed by extensive discussions among all members in attendance ( Alguire, 1998; Aronson, 2017; Bhattacharya, 2017; Linzer, 1987; McGlacken-Byrne *et al.*, 2020). This format is known for its educational benefits, including critical thinking and improved appraisal skills, increased research awareness, knowledge of methodology, and study design ( McGlacken-Byrne *et al.*, 2020). Despite these benefits, the classic journal club greatly resembles didactic lectures and seminars. This format has many pitfalls including but not limited to passive listening. In addition, journal clubs facilitated by faculty members may be more hierarchal where members with more experience have the tendency to dominate the discussions ( Rosenthal & Rosenthal, 2017). Furthermore, with the recent COVID-19 pandemic, face-to-face meetings are difficult to facilitate because of health and safety protocols that include physical distancing and avoidance of mass gatherings in an enclosed space. With the rise of available virtual platforms where journal clubs may be conducted allows for a more flexible approach in terms of delivery and execution. Different approaches have evolved overtime to encourage active participation and improve engagements. However, most of these approaches remain similar to the traditional approach and thus retain its pitfalls. Furthermore, many view the journal clubs as a routine academic requirement further hampering participant engagement and individual growth potential ( Holmes *et al.*, 2015).

The interactive journal club is designed to utilize a new approach in appraising research in order to maximize the benefits of the traditional journal club. In this new approach, the participants are actively involved in a structured process of critical data appraisal rather than just being passive listeners. Its main goals are centered on an engaged, participatory, and an overall enjoyable medical learning experience. This revision uses the active learning approach to maximize individual participation, opportunities for critical thinking, better communication skills, active reflection, and self-confidence ( Graffam, 2007; Prince, 2004). The interactive journal club does not require substantial training and expertise. Briefly, after choosing the topic and journal article, the Designated Leader (DL) directs the flow of discussion with a set of guide questions that aims to elicit individual responses from participants. All participants are viewing the article for the first time and are prompted to review the title, methodology, raw data and results. Participants must provide their own interpretation and critique of the article and are challenged to be more adept at study design, data analysis, and presentation. In most studies utilizing the interactive journal club format, participants have feedbacked that they look forward to each session as these are enjoyable, relevant, and beneficial ( Rosenthal & Rosenthal, 2017).

 In our institution, our endocrinology fellows are tasked to present two journal articles every week within a span of one hour using the traditional journal club format where one trainee will present an article usually on a landmark trial while the rest of the participants are passive learners. Hence, in this study, we applied the interactive journal club format to the same participants and assessed the impact of the interactive journal club through the analysis of session recordings and participant feedback.

## Methods

### Ethical considerations

As the sessions were part of some innovative changes in our training program, we were not performing a study and did not intend to share the findings originally, therefore ethical approval was not sought. The registration for this study has been approved by the Research Grants Administration Office, University of the Philippines Manila (Registration Number RGAO 2023-1025). All participants in the study provided informed consent for video recording and provision of participant feedback after each session. All the data were completely de-identified. The study was conducted in accordance to the Declaration of Helsinki. The manuscript was written in accordance with the STROBE guidelines for reporting observational studies.

### Interactive journal club sessions

Briefly, we conducted a series of interactive journal club sessions within a four-week span, one per each week *via* a virtual platform (Zoom) from October 1 to 31, 2021 among the fellows-in-training of the Division of Endocrinology, Diabetes and Metabolism of the Philippine General Hospital, University of the Philippines Manila. The participants were the same in all sessions conducted (11 endocrinology fellows and one faculty member). All participants except the faculty member were grouped into three teams with three to four members. Prior to the interactive session, each team met on their own to decide and discuss the journal article to be presented to the participants. The choice of the journal article was decided by members of each team by consensus voting.

We used the steps and processes provided by Rosenthal J and Rosenthal KS (2017) in our interactive sessions. Each session was facilitated by one team, with the most senior trainee acting as the DL for that session. The teams rotated as facilitators of the interactive sessions. The same presentation template used by the first team was used throughout the four sessions. The faculty member remained an active participant in all sessions, observed the interactions of the group, and provided feedback after each session. Each session was recorded *via* Zoom platform following consent from all the participants. Following each session, participant feedback was obtained, tabulated and compared to the traditional journal club format.

### Data collection methods

Prior to data collection, verbal consent for data gathering and video/audio recording were secured from each participant during the course orientation. Data were recorded from October 1 to 31, 2021 during our weekly interactive journal club sessions. Each session was recorded using the Zoom platform and timed to determine the total duration of the activity. After finishing all sessions, the participants were gathered and their insights and opinions regarding the new format were sought.

### Data processing

All information and insights from each session were tabulated and presented as narratives in Microsoft Word. All identifiers were removed from the summary statements. All video and audio recordings were kept in a secured and password protected Google Drive with only the study authors having access. The cloud data will be stored only until five years from the time of upload and will be permanently deleted afterwards.

### Data analysis

The researchers identified and narrated the inferences using a comparative approach between a lecture-driven model (traditional approach) *versus* an adult interactive learning model (interactive approach).

### Reflexivity statement

We were both former trainees who underwent the traditional models of training for our weekly conferences. On one hand, we initially had hesitations to adopt new approaches in our curriculum as we were all very used to our usual routines. As the first and oldest training institution in Endocrinology, Diabetes and Metabolism in the Philippines, our traditional approaches have been utilized for decades. Overcoming these great barriers would require tantamount courage and determination. On the other hand, as active proponents of innovative changes and use of technological advances in medical education, we were biased towards its positive reception and acceptance amongst our trainees. Therefore, it was exciting to be able to explore this new approach. However, we faced several challenges in adopting this new approach. First, there was skepticism and doubt arising from ourselves as well as members of the group if this could actually work. Second, we did not have baseline knowledge on this approach, and venturing towards uncharted territory can be daunting even for an experienced and multi-awarded educator and teacher of more than two decades (Dr. Iris Isip-Tan). Lastly, adopting this approach during the height of the COVID-19 pandemic also contributed to our further uncertainty on acceptability and utility. Despite all these, we persisted as we wanted to understand and evaluate this new approach in critical appraisal and medical learning. To ensure objectivity and minimize bias during the compilation of participant feedback, we accounted both positive and negative comments solely from the study participants while we inhibited ourselves from including our thoughts and insights.

## Results

A summary of our interactive journal club sessions, which utilized the following works ( Lanzolla *et al.*, 2021; Painter *et al.*, 2022; Schietroma *et al.*, 2013; Siricharoenthai & Phupong, 2020), as outlined in Table 1. During the start of the first session all participants were hesitant to spearhead the discussion. We encountered multiple times of pauses before a volunteer stepped in to continue the flow of discussion and the DL had to call the less active members to equalize the contributions from all participants. The first session was also the longest in terms of duration due to the participant’s unfamiliarity. During our subsequent sessions, as the participants became more comfortable and familiar with the setup, we observed a more spontaneous flow of discussion and more active participation compared to the previous session. Sessions two to four finished in a lesser span of time.

**Table 1.** Summary of interactive journal club sessions.

Topics	Total Time (minutes)
Session 1 Diagnostic accuracy of HbA1c in detecting gestational diabetes mellitus ( Siricharoenthai & Phupong, 2020)	83
Session 2 Dexamethasone for the Prevention of Recurrent Laryngeal Nerve Palsy and Other Complications After Thyroid Surgery: A Randomized Double-blind Placebo-Controlled Trial ( Schietroma *et al.*, 2013)	64
Session 3 Statins for Graves' orbitopathy (STAGO): a phase 2, open-label, adaptive, single center, randomized clinical trial ( Lanzolla *et al.*, 2021)	59
Session 4 Lower versus Higher Glycemic Criteria for Diagnosis of Gestational Diabetes ( Painter *et al.*, 2022)	65
	Average: 67.75

At the end of the four interactive journal club sessions, verbal feedback was obtained from each participant and tabulated as to how it compared to the traditional journal clubs that they attend on a weekly basis. Our case study demonstrated that the interactive journal club format was found to be more fun and proactive, promotes equality among participants, involves active participation, encourages critical thinking and increases self-esteem and confidence. Emphasis was also placed on learning from input of other colleagues. The participants are also more likely to apply the learnings in their own daily practice ( Table 2). Our findings are consistent with the published framework and template exploring differences between the traditional approaches (lecture-driven models) *versus* interactive approaches (adult interactive education models) ( Rosenthal & Rosenthal, 2017). Furthermore, in our case study, all participants did not undergo training prior to the sessions. This was also supported by existing literature that while an interactive approach encourages critical thinking, analysis, reflection, and synthesis of information, no significant amount of training, revision and preparation of materials are needed to help facilitate and achieve its goals ( Rosenthal & Rosenthal, 2017). Overall, our case study findings support the notion that an interactive journal club allows the retention of the spirit and pivotal role of the traditional journal club tradition in medicine, while answering the call for modern approaches to medical education.

**Table 2.** Comparison of experiences between the interactive journal club and traditional journal club of the participants.

Traditional Journal Club	Interactive Journal Club
• Monotonous • More hierarchical • Passive listener • Tendency to accept what is presented • Less opportunity to interact with colleagues • Higher hesitancy to ask questions	• More fun and proactive • More equality among participants • Active participation • Encourages critical thinking • Learning from inputs of colleagues • Increases self-esteem and confidence • Application in own daily practice

## Discussion

The interactive journal club format provides a systematic, direct and easy to follow approach for reviewing a journal article. It is able to promote active participation where all participants become involved in the discussion of the title, methods and relevant data as compared to the traditional format where participants are simply passive listeners. Each session starts and ends with particular emphasis to the article’s title to clarify the objectives, study results and significant learning points. From the start of the process, it already provides the group with the opportunity of problem solving and critical thinking. Data analysis allows each participant to assess the accuracy of the information and to formulate their own conclusions as compared to ready acceptance of the author’s conclusion. Towards the end of each session, by going back to the title once more, the participants then evaluate the article’s success towards reaching the goals as is suggested in their title. Overall, this process allows all participants to interact with one another through in-depth discussion and critical inquiry ( Rosenthal & Rosenthal, 2017).

 The participants are given the opportunity to review the raw data by applying their previous knowledge of research design and methodology. This allows them to become familiar and comfortable in evaluating research methodology, study design, formal analysis, and data presentation. Participants are also given the opportunity to reflect individually and as a group on the available information, their level of understanding and the discussion proper. Furthermore, they can observe how others assess and evaluate information, challenge their own and each other’s thoughts, provide and receive feedback, and come up with their own understanding of the concepts. Individual reflections and shared understanding among participants enhance the learning experience and enrich each member’s understanding. The participative and interactive learning process is integral to the interactive journal club method further strengthening its appeal to adult and professional learners ( Holmes *et al.*, 2015; Rosenthal & Rosenthal, 2017).

 A major portion of the interactive approach is the emphasis given to the process of content analysis. The DL facilitates and guides all participants with the set of guide questions during critical analysis. With time, this process becomes repetitive and consistent across all sessions to provide a reliable framework for data collection and review that extends beyond the journal club itself ( Rosenthal & Rosenthal, 2017).

In all our sessions, all participants came to the conclusion that the articles we discussed were not able to prove what was initially described in the title. They found that most authors often overstate and force their findings into conclusions while some do not accurately report the results in the text. Our participants were also able to identify study limitations, gaps in methodology, and statistical analysis, all of which were not explicitly stated in the main text but could compromise the validity and applicability.

One of the major advantages of the interactive format is its flexibility and adaptability to a diverse group of learners while keeping the basic approach repetitive and consistent ( Rosenthal & Rosenthal, 2017). This approach has, in fact, been used by medical students, residents, clinical fellows and even faculty members. Sessions can be scheduled during designated class periods or during working lunches. It can be done in a face-to-face setting with small and large groups in meeting rooms, large auditoriums or *via* online meeting platforms.

 Our case study has several limitations. First, we are limited only to a single academic center and a small number of trainees. This may limit the generalizability of our findings to other training and academic institutions. Second, we conducted the sessions as part of the training requirements supervised by a senior consultant faculty and this could have affected the level of participation and enthusiasm of all members in the group. Third, all participants are familiar with each other and this could have facilitated better communication and coordination during the sessions. Lastly, this case study was conducted at the height of the COVID-19 pandemic where most academic meetings were conducted online and this could also have an effect on participant involvement as compared to a face-to-face session. These limitations have to be considered when interpreting our findings.

Overall, the participants have pointed out that the interactive approach makes learning more fun and an overall proactive experience. The approach was able to allow more critical thinking and processing of information as compared to being a passive recipient. The open and free-for-all nature of group participation has increased their self-esteem and confidence resulting in increased active participation. Others have indicated that the interactive journal club allowed them to learn from the inputs of other participants that they themselves were not able to extract alone, they also had better retention of the information discussed in each article, while the rest have pointed out that they will utilize this approach in their future practice as well as apply this when they go back to their respective provinces after completion of training.

## Conclusion

Modern medical education has evolved through the years by shifting from the traditional lecture-type, top to down models to more interactive and reflective adult education models in order to enhance critical thinking and understanding. The interactive journal club format provides an avenue to revolutionize the traditional journal club by incorporating principles that encourage active participation, critical analysis, self-reflection, processing of information and participant feedback without the need for extensive training, revision and preparation. Through this learning model, traditional approaches are transformed from passive presentations of recent developments in medicine into an interactive discussion while allowing the retention of the spirit and essence of a traditional journal club.

## Data Availability

The underlying data cannot be shared due to privacy concerns; however sessions were recorded and can be provided on request. The reviewers and readers may contact the corresponding author via email ( hcchiu@alum.up.edu.ph) for the copy of the videos after providing their complete academic affiliations and verified academic email addresses. No part of the said videos are allowed to be shared outside the purpose for which it was requested.
