# Supplementary material for: Application and impact assessment of an interactive journal club format among endocrinology fellows-in-training in a tertiary hospital academic center: a case study
Source: MedEdPublish (2016). 2023 Sep 8;13:111. [Version 1] doi: 10.12688/mep.19740.1 (PMC10844799; doi:10.12688/mep.19740.1)
Supplement: Peer review history

# Reviewer report: Reviewer response for version 1

**DOI:** 10.21956/mep.21150.r34769

**Published:** 2024-02-05

**Authors:** Daveric Pagsisihan

**Affiliations:** 1 De La Salle Health Sciences Institute, Cavite, Philippines

The topic of the case study is very timely as more educational interactions are given with a virtual option. The process of an interactive journal club improves how the assessment and appraisal of articles are conducted by trainees and the interaction between participants. It also encourages active participation and a lively engagement with the attendees. It was proven to be an easy activity, with no special skills needed. It has made the process enjoyable for all the participants.

 Few things that need to be clarified. First, is there any rationale for grouping the participants into 3? The process described by Rosenthal did not state the grouping of the participants, especially if there are only 11 participants (smaller group).

 Second, the DL is always a senior trainee of the assigned group. What roles were given to the other 2 or 3 lead group members?

 Lastly, aside from multiple pauses during the session, what other technical and non-technical difficulties were encountered?

Is the case presented with sufficient detail to be useful for teaching or other practitioners?

Yes

Is the work clearly and accurately presented and does it cite the current literature?

Yes

If applicable, is the statistical analysis and its interpretation appropriate?

Not applicable

Is the case’s background and context in sufficient detail?

Yes

Are all the source data underlying the results available to ensure full reproducibility?

Partly

Are the conclusions drawn adequately supported by the results?

Yes

Reviewer Expertise:

Epidemiologic/observational type of research; this is the first time I am reviewing an academic/medical education-related paper.

I confirm that I have read this submission and believe that I have an appropriate level of expertise to confirm that it is of an acceptable scientific standard.

# Reviewer report: Reviewer response for version 1

**DOI:** 10.21956/mep.21150.r34770

**Published:** 2023-10-17

**Authors:** Elaine Cunanan

**Affiliations:** 1 University of Santo Tomas, Manila, Metro Manila, Philippines

The authors aimed to qualitatively assess whether a more interactive approach (adapted from the "interactive journal club" format by Rosenthal et al. 2017) enhances adult learning (in terms of boosting participation and making the activity more enjoyable) compared to traditional critical appraisal reporting of a journal article via an online setting.

 Indicate how the following sources of bias were addressed:

1. Hawthorne effect
2. Interviewer bias
3. Interviewee response bias

The responses seem to be all positive. Is there a possible downside?

 Provide a clear process flow (diagram) for the activity to be replicable by interested readers.

Is the case presented with sufficient detail to be useful for teaching or other practitioners?

Partly

Is the work clearly and accurately presented and does it cite the current literature?

Yes

If applicable, is the statistical analysis and its interpretation appropriate?

Not applicable

Is the case’s background and context in sufficient detail?

Yes

Are all the source data underlying the results available to ensure full reproducibility?

Partly

Are the conclusions drawn adequately supported by the results?

Yes

Reviewer Expertise:

medical education

I confirm that I have read this submission and believe that I have an appropriate level of expertise to confirm that it is of an acceptable scientific standard.
